# Phenotype and genotype analyses of 21 Chinese patients with Dent disease

**DOI:** 10.7555/JBR.38.20240183

**Published:** 2025-05-27

**Authors:** Ruochen Che, Yuwen Cai, Wei Zhou, Sanlong Zhao, Songming Huang

**Affiliations:** 1 Department of Nephrology, Children's Hospital of Nanjing Medical University, Nanjing, Jiangsu 210029, China; 2 Nanjing Key Laboratory of Pediatrics, Children's Hospital of Nanjing Medical University, Nanjing, Jiangsu 210029, China; 3 Jiangsu Key Laboratory of Pediatrics, Nanjing Medical University, Nanjing, Jiangsu 211166, China

**Keywords:** Dent disease, *CLCN5* gene, *OCRL* gene, low-molecular-weight proteinuria, children, X-chromosome inactivation

## Abstract

Dent disease is a rare X-linked recessively inherited renal tubulopathy, caused by variants in the *CLCN5* (Dent disease type 1, DD1) and *OCRL* (Dent disease type 2, DD2) genes, and characterized by low molecular weight proteinuria, hypercalciuria, microscopic hematuria, or nephrocalcinosis. In the current study, we collected and analyzed clinical data and genetic testing results of 21 children diagnosed with Dent disease, who were hospitalized at the Department of Nephrology, Children's Hospital of Nanjing Medical University between January 2014 and August 2023, aiming to assist in the early diagnosis and treatment of these patients. Among the 21 patients, 16 (76.19%) had *CLCN5* variants, and five (23.81%) had *OCRL* variants, and four of the variants were novel. All patients presented with low molecular weight proteinuria, 14 (66.67%) of whom had nephrotic-range proteinuria. Eight patients underwent renal biopsies because of diagnostic uncertainty. We transfected the novel *CLCN5* missense variant (p.G222R) and *OCRL* missense variant (p.I371T) plasmids into both HEK293 and HK-2 cells and found a significantly lower expression of the OCRL1 protein in the transfected cells than in the wild-type cells (*P* < 0.05). Moreover, we observed an extremely skewed X-chromosome inactivation pattern in a female patient carrying the same novel *CLCN5* variant, as assessed by the human androgen receptor gene assay. These findings provide insight into the clinical characteristics of Dent disease in Chinese patients and may shed light on its pathogenesis.

## Introduction

Dent disease is a rare X-linked recessively inherited tubulopathy characterized clinically by low molecular weight proteinuria (LMWP), hypercalciuria, nephrocalcinosis, kidney stones, and progressive renal failure in some patients. In approximately 60% of patients, the disease is caused by inactivating variants in the *CLCN5* gene (Online Mendelian Inheritance in Man No. 300009; Dent disease type 1, DD1) located on the short arm of the X chromosome (Xp11.22). Another 15%–20% of patients have variants in the *OCRL* gene (Online Mendelian Inheritance in Man No. 300535; Dent disease type 2, DD2) located on the long arm of the X chromosome (Xq25)^[1–3]^. The remaining cases are caused by genetic variants that have not yet been identified.

Chloride voltage-gated channel 5 (CLC-5), encoded by *CLCN5*, is an α-chloride channel protein expressed in proximal tubules, thick ascending limbs, and the intercalated cells of collecting ducts^[4]^. Variants in the *CLCN5* gene cause truncation or absence of the CLC-5 protein, which affects both chloride conductance and antiporter functions in proximal tubular epithelial cells, thus impairing the acidification function of vesicles and leading to compromised endocytosis in these cells^[5]^. This disruption may trigger abnormalities in a variety of clinical phenotypes observed in patients with Dent disease.

The inositol polyphosphate 5-phosphatase OCRL1 is a transmembrane protein encoded by *OCRL* and mainly distributed in the Golgi complex, lysosomes, and early endosomes of proximal tubule epithelial cells. OCRL1 hydrolyzes phosphatidylinositol 4,5-bisphosphate (PIP2) *via* its 5-phosphatase domain and regulates transient receptor potential cation channel subfamily V member 6 (TRPV6)-mediated Ca^2+^ transport, through Rab-binding activity, without altering TRPV6 membrane abundance. OCRL1 also suppresses TRPV6-mediated Ca^2+^ transport by modulating PIP2 levels essential for TRPV6 function, independent of TRPV6 protein expression or surface localization^[6]^.

As a rare disease, the comprehensive understanding of Dent disease remains incomplete. Because of the insufficient knowledge of this condition among pediatricians and the absence of effective clinical screening tools, potential misdiagnosis and improper treatment of the disease may occur. The current study retrospectively examined clinical data and genetic testing results of 21 children diagnosed with Dent disease at the Department of Nephrology, Children's Hospital of Nanjing Medical University between January 2014 and August 2023, aiming to enhance awareness and understanding of this disease.

## Materials and methods

### Study population

The 21 patients with Dent disease were diagnosed according to the following diagnostic criteria^[7]^: (1) The presence of LMWP referred to a urine protein electrophoresis of LMWP ratio > 50% or urine alpha-1-microglobulin to microalbumin ratio > 1^[8]^; (2) Pathogenic variants were found in the *CLCN5* or *OCRL* gene analysis; (3) With or without one of the following symptoms in patients: hypercalciuria, hematuria, kidney stones, urinary calcium salt accumulation, hypophosphatemia, and renal dysfunction; and (4) Any disorders with proximal tubule dysfunctions because of other known causes were excluded, such as drug toxicity, infections, autoimmune diseases, congenital disorders (*e.g.*, Lowe syndrome), and systemic metabolic diseases (*e.g.*, Wilson's disease and cystinosis). Hypercalciuria was determined using previously reported age-specific reference values^[9]^. Nephrotic-range proteinuria (NP) was determined as the first morning or 24-h protein-creatinine ratio ≥ 2 g/g (or 200 mg/mmol or ≥ 3+ dipstick) according to the latest guidelines of KDIGO 2021. Hypoalbuminemia was determined as serum albumin < 25 g/L. Hypophosphatemia was determined as the serum phosphorus concentration < 0.8 mmol/L. Renal biopsy was performed in eight of the 21 cases at the Children's Hospital of Nanjing Medical University under B-ultrasound guidance, and the samples were examined by light microscopy, immunofluorescence, and electron microscopy, respectively.

Additionally, a comprehensive literature search on Dent disease was carried out across a total of seven databases, including three English databases (MEDLINE, Cochrane Library, and EMBASE). Relevant studies were identified through the use of search terms such as "Dent disease", "*CLCN5*", and "*OCRL*". The inclusion criteria for these studies were as follows: (1) publication in English; (2) study participants were children under the age of 18; and (3) clinical symptoms including NP.

The current study was carried out in accordance with the World Medical Association's Helsinki Declaration and was approved by the Institutional Review Board of Children's Hospital of Nanjing Medical University (Approval No. 201710001-1). Written informed consent was obtained from the parents.

### DNA preparation

Genomic DNA was isolated from the peripheral blood of the participants using the DNA isolation kit (Cat. #DP423, Tiangen, Beijing, China) according to the manufacturer's protocol. The genome was sheared into fragments, and then hybridized with the xGenExome Research Panel v2.0 probe sequence capture array from Integrated Device Technology to enrich the exonic region. All identified variants were annotated using the 1000 Genomes Project (Chinese), dbSNP, Genome Aggregation Database (gnomAD), and ExAC databases. Variants with a minor allele frequency higher than 5% were filtered out. Finally, candidate variants were assessed using the American College of Medical Genetics and Genomics (ACMG) criteria and further validated by direct Sanger sequencing.

### Direct Sanger sequencing of the *CLCN5* and *OCRL* genes

All the primer pairs were designed to amplify the exons of the *CLCN5* and *OCRL* genes (***Supplementary Table 1***, available online). Polymerase chain reaction (PCR) was performed in a 25 μL reaction mixture containing 1.5 μL primer, 2.0 μL template DNA, 2.5 μL MgCl_2_, 1.2 μL 2.5 mmol/L dNTPs, 2.5 μL of 10× Taq buffer containing (NH_4_)_2_SO_4_, and 0.2 μL Taq DNA polymerase. The PCR cycling conditions included a predenaturation at 94 ℃ for 5 min, denaturation at 94 ℃ for 30 s, annealing at 59 ℃ for 30 s, and extension at 72 ℃ for 30 s. The last three steps were repeated for 35 cycles, followed by a final extension at 72 ℃ for 5 min. The PCR products were gel- and column-purified and then sequenced using BigDye Terminator (Applied Biosystems, Foster City, CA, USA) and ABI373 sequencer (Applied Biosystems). The cDNA reference number in the current study is NM_000084.4 for *CLCN5* and NM_000276.3 for *OCRL*.

### Construction of plasmid vectors

The cDNAs of human *CLCN5* (h*CLCN5*) and human *OCRL* (h*OCRL*) were constructed using PCR, with h*CLCN5* and h*OCRL* cloned into the pcDNA3.1-3×Flag vector using the Clon Express Entry One Step Cloning Kit (Vazyme, Nanjing, China). Mutations in *CLCN5* and *OCRL* were introduced *via* the PCR-based DpnI-treatment method, employing the Mut Express Ⅱ Fast Mutagenesis Kit V2 (Vazyme) and appropriate primers (***Supplementary Table 1***). Plasmids were generated from both wild-type (WT) and mutant pcDNA3.1-3×Flag-h*CLCN5* and pcDNA3.1-3×Flag-h*OCRL* using PCR mutagenesis. The complete coding sequences of all constructs were verified through sequencing.

### Cell culture and transfection

Both HEK293 and HK-2 cells were transiently transfected with either WT (pcDNA3.1-3×Flag-WT DNA) or mutant (pcDNA3.1-3×Flag-h*CLCN5*-G222R or pcDNA3.1-3×Flag-h*OCRL*-I371T) plasmids. Briefly, 24 h before transfection, HEK293 or HK-2 cells were seeded in six-well plates with 2 mL of Dulbecco's Modified Eagle's Medium (DMEM) per well, and incubated at 37 ℃ in a 5% CO_2_ atmosphere. When the cells reached 50%–70% confluence, they were transfected with each purified plasmid using Lipo2000 (Cat. #11668027, Invitrogen, Carlsbad, CA, USA) according to the manufacturer's protocol. The final concentration of each plasmid post-transfection was 0.01 μg/μL. The transfection was carried out for 4 h with 2 μg of each plasmid, and the cells were subsequently cultured and harvested after 24 h.

### Western blotting analysis

A lysis buffer containing protease inhibitors was used to generate whole-cell lysates from HEK293 or HK-2 cells after they were rapidly washed with ice-cold phosphate-buffered saline. Bovine serum albumin was used as a standard in the Micro BCA protein assay kit (Thermo Fisher Scientific, Waltham, MA, USA). The total protein (20 μg) was separated by 10% sodium dodecyl sulfate polyacrylamide gel electrophoresis and transferred to polyvinylidene fluoride membranes. We blocked the membranes in TBST (0.1% Tween 20 in TBS) containing 5% nonfat milk at room temperature for 1 h and incubated them with primary antibodies against Flag (1∶3000; Cat. #A2220, Sigma-Aldrich, St. Louis, MO, USA) or GAPDH (1∶3000; Cat. #60004-1-Ig, Proteintech, Wuhan, China) at 4 ℃ overnight. After incubation with horseradish peroxidase-labeled secondary antibodies (1∶5000; Cat. #SA00001-1, Proteintech) at room temperature for 1 h, chemiluminescence (Cat. #32106, Thermo Fisher Scientific) was used to visualize protein bands.

### X-chromosome inactivation (XCI) detection

The XCI pattern was detected by the human androgen receptor gene (HUMARA) assay^[10]^. Since the inactivated X chromosome is methylated and the active X chromosome is unmethylated, for each DNA sample, two PCR reactions were performed. In one reaction, the template contained DNA digested with the methylation-sensitive restriction enzyme HpaII (Cat. #R0175L, New England Biolabs), whereas the other reaction contained undigested genomic DNA. PCR products were electrophoresed and analyzed. XCI was judged according to (d1/u1)/(d1/u1 + d2/u2), where d1 and d2 represent the subject's digested allele (+HpaII) and u1 and u2 represent the undigested allele (−HpaII). When the enzymatic cleavage is incomplete, amplified products of the *MIC2* gene (located at the ends of the short arms of chromosomes X and Y) can be observed. Therefore, *MIC2* was used as a reference to evaluate the digestion efficiency. The XCI pattern was defined as random (from 50∶50 to 59∶41), mildly skewed (from 65∶35 to 79∶21), or extremely skewed (more than 80∶10).

### Statistical analysis

Figures were generated using Illustrator for Biological Sequences 1.0 and Pymol 2.1.1. The statistical software Prism 10.0 was used for statistical analyses. Relative protein expression levels were presented as the mean ± standard error of the mean, and differences between two groups were compared using unpaired Student's *t*-tests. The Chi-square test was used to compare proportions between groups. *P* < 0.05 was considered statistically significant. The unreported variants were evaluated using the *in silico* prediction programs, including Polymorphism Phenotyping v2 (PolyPhen-2, https://genetics.bwh.harvard.edu), SIFT (https://sift.bii.a-star.edu), and Consurf (https://consurf.tau.ac.il).

## Results

### Cohort clinical and renal pathological characteristics of the patients

The 21 children diagnosed with Dent disease comprised 11 familial cases and nine *de novo* cases, with a male-to-female ratio of 20∶1. The median age at diagnosis was 50 months (range, 1–164 months), and all patients had LMWP. Clinical details of the 21 children with Dent disease are presented in ***Table 1***, and further detailed phenotyping is shown in ***Supplementary Table 2*** (available online). In addition, one of the patients (patient 12, P12) developed chronic kidney disease at an early stage [eGFR 94 mL/(min·1.73 m^2^)] and progressed to the fourth stage of chronic kidney disease within six years. Despite significantly elevated levels of parathyroid hormone and alkaline phosphatase, the blood phosphorus of the patient was decreased, suggesting that he had secondary rickets. Pituitary magnetic resonance imaging showed the inferior position of the cerebellar tonsils, which was approximately 3 mm below the foramen magnum.

**Table 1:** Characteristics of 21 pediatric patients with Dent disease

Characteristics	All patients (*n*=21)
Sex (male/female, *n*)	20/1
Familial/sporadic cases (*n*)	11/9
Age at diagnosis (months)	
Median age	50
Range	1–164
Height (*Z*-score) at diagnosis (mean ± SD)	−0.142±1.968
Microscopic hematuria [*n* (%)]	5 (23.81%)
Hypercalciuria [*n* (%)]	8 (38.10%)
NP [*n* (%)]	14 (66.67%)
LMWP [*n* (%)]	21 (100%)
Nephrocalcinosis [*n* (%)]	10 (47.62%)
Gene (*CLCN5*/*OCRL*, *n*)	16/5
Kidney biopsy (*n*)	8
FSGS	4
FGGS	3
MCD	1
Abbreviations: NP, nephrotic-range proteinuria; LMWP, low-molecular-weight proteinuria; FSGS, focal segmental glomerulosclerosis; FGGS, focal global glomerulosclerosis; MCD, minimal change disease; SD, standard deviation.	

Interestingly, patient 4 (P4) was the only female child who developed symptoms at the age of four years and 11 months, and her urine protein test returned positive. Following treatment with traditional Chinese medicine, her urine protein levels did not normalize, and the urine alpha-1-microglobulin value was more than five times higher than the normal value, indicating LMWP. However, she did not exhibit the typical symptoms of Dent disease, such as hypercalciuria, nephrocalcinosis, or microscopic hematuria. Additionally, her father had a history of proteinuria and kidney stones. Patient 18 (P18) was a male child who showed typical LMWP but lacked typical symptoms such as hypercalciuria and nephrocalcinosis. The pathological result of the renal biopsy was focal global glomerulosclerosis (FGGS).

Kidney biopsy was performed in eight patients, including five with DD1 and three with DD2. The renal biopsy suggested that patient 6 (P6) had FGGS, mild mesangial matrix proliferation, focal tubular atrophy, and mild interstitial fibrous tissue proliferation, while patient 9 (P9) had mild mesangial stromal proliferation, fibrosis of Bowman's capsule in one glomerulus, and focal atrophy of the periglomerular tubules with focal interstitial fibrosis. The first renal biopsy of P12 showed chronic interstitial lesions with glomerulosclerosis, and six years later, the second renal biopsy results revealed severe focal segmental glomerulosclerosis (FSGS), severe chronic lesions in 80% of the tubulointerstitium, massive inflammatory cell infiltration in the interstitium, and arterial hyalinosis. Patient 14 (P14) exhibited FGGS with focal glomerular atrophy and focal fibrosis, while patient 15 (P15) was diagnosed with FSGS accompanied by renal tubular damage. Renal biopsy results indicated FGGS for both patient 18 (P18) and patient 21 (P21), while patient 19 (P19) was diagnosed with minimal change disease (MCD), with the renal tubulointerstitium appearing normal. Electron microscopy revealed mild fusion of the foot process segments in all eight children. Specific renal biopsy pathology results are shown in ***Table 2*** and ***Supplementary Fig. 1*** (pathology for child P12 was only verbally reported; report available online).

**Table 2:** Renal manifestations in eight children with Dent disease

Patient	P6	P9	P12	P14	P15	P18	P19	P21
Age at biopsy (years)	1.3	12.1	13	5.7	13.9	12.9	10.5	13.6
Glomerulus (*n*)								
Glomeruli	53	6	NA	22	42	34	16	10
Sclerotic glomeruli	2	0	NA	1	4	1	0	1
Foot process fusion	+	+	NA	+	+	+	**−**	**−**
Tubules								
Tubular atrophy	+ 2%	+ 3%	+	+	+ 5%	**−**	−	**−**
Interstitial fibrosis	+ 2%	+ 3%	+ 80%	+	+	**−**	**−**	**−**
Immunofluorescence	**−**	**−**	IgM	**−**	**−**	**−**	**−**	**−**
Pathological diagnosis	FGGS	FSGS	FSGS	FGGS	FSGS	FGGS	MCD	FGGS
Glomeruli, sclerotic glomeruli, and foot process fusion are quantified as absolute counts per biopsy sample. Tubular atrophy and interstitial fibrosis are graded semi-quantitatively (mild/moderate/severe) based on histological extent.Abbreviations: P, patient; NA, not applicable; +, positive; **−**, negative; FGGS, focal global glomerulosclerosis; FSGS, focal segmental glomerulosclerosis; MCD, minimal change disease.								

### Variants detected in the *CLCN5* and *OCRL* genes

**Figure 1:**
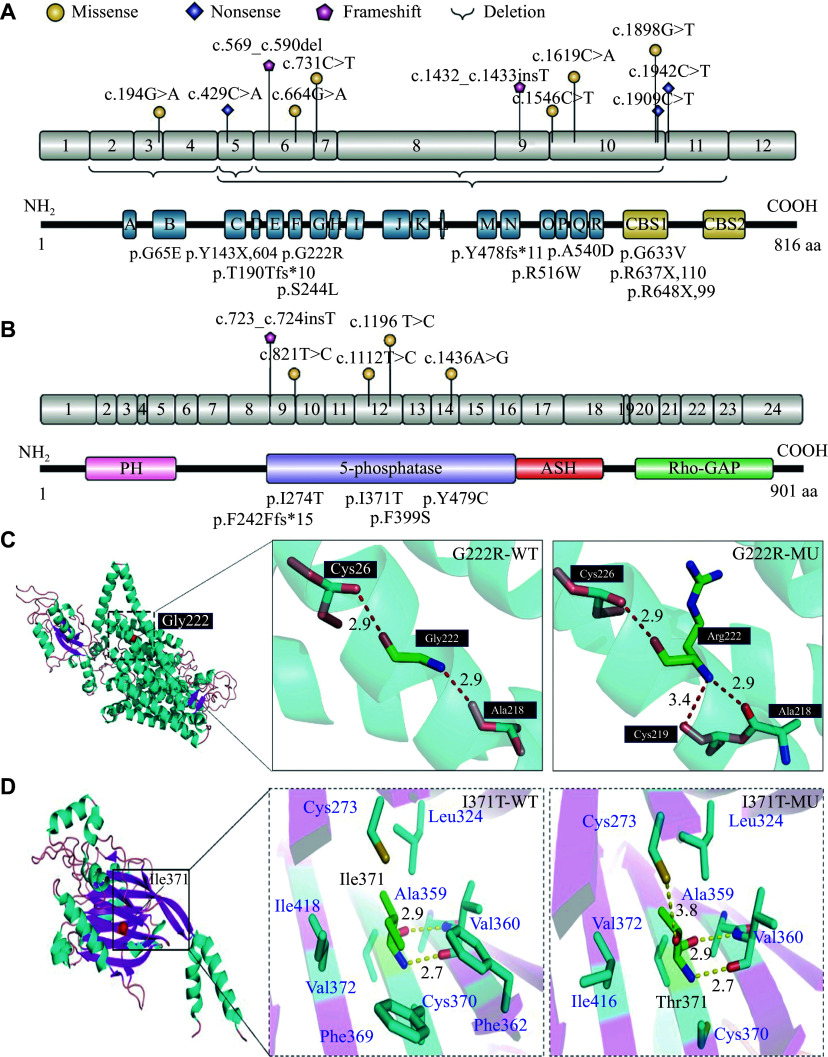
Exon structure of the *CLCN5* and *OCRL* genes with geometric shapes indicating the relative positions of different types of mutations. A: The deep blue rectangle unit encodes the 18 alpha-helices (from A to R) of CLC-5 protein, and the yellow rectangle unit describes the cystathionine beta-synthase (CBS) domain of the CLC-5 protein. Amino acids (located in helices D, F, N, and R) forming the chloride selectivity filter (33, 34), and helices H, I, P, and Q are involved in the formation of the interface (24, 33). B: The pink rectangle unit encodes the pleckstrin homology (PH)-like domain; the green rectangle unit encodes the 5-phosphatase catalytic domain; the red rectangle unit encodes the ASPM, SPD-2, Hydin (ASH) domain; and the purple rectangle unit encodes the Rho GAP-like domain of the OCRL1 protein. C: Three-dimensional model of CLC-5 based on the AlphaFold entry AF-P51795-F1 (from amino acids 1 to 746) showing the location of the *CLCN5* mutations. D: Three-dimensional model of OCRL1 based on the PBD entry 4CMN (from amino acids 215 to 560) showing the location of the *OCRL* variation. They are all viewed as monomers, and the mutated residues are labeled in white font.

All 21 patients and their parents had their DNA extracted from peripheral blood for genetic analysis. Targeted-panel sequencing (gene panel) was performed on 13 cases, while whole-exome sequencing was performed on eight cases. In addition, patient 1 (P1) was also screened by a mitochondrial panel. The results showed that 76.19% of cases carried variants in the *CLCN5* gene, while the remaining cases carried variants in the *OCRL* gene, but the mitochondrial panel of P1 was normal. Upon sequence analysis of the *CLCN5* gene in 16 patients, 15 variants were identified in exons 3–11. These 16 variants included missense (7, 43.75%), deletion (4, 25.00%), nonsense (3, 18.75%), and frameshift (2, 12.50%). Overall, 21 variants were distributed throughout the coding sequences of *CLCN5* and *OCRL*, as well as in the corresponding protein structures (***Fig. 1***). Three variants in the *CLCN5* gene (deletion of exon 5, deletion of exons 2–4, and G222R), as well as one missense variant (I371T) in the *OCRL* gene, were novel and had not been previously reported. According to the ACMG guidelines, the deletion of *CLCN5* exon 5 was considered likely pathogenic, while the deletion of *CLCN5* exons 2–4 was classified as pathogenic. Prediction software was used to assess the pathogenicity of two novel missense variants, and they were both predicted to be deleterious. Using PyMOL software, we predicted the structural changes in both proteins resulting from the missense variants. The variants altered both the number and orientation of hydrogen bonds with the surrounding structure. The detailed genetic variants are summarized in ***Supplementary Table 2***.

**Figure 2:**
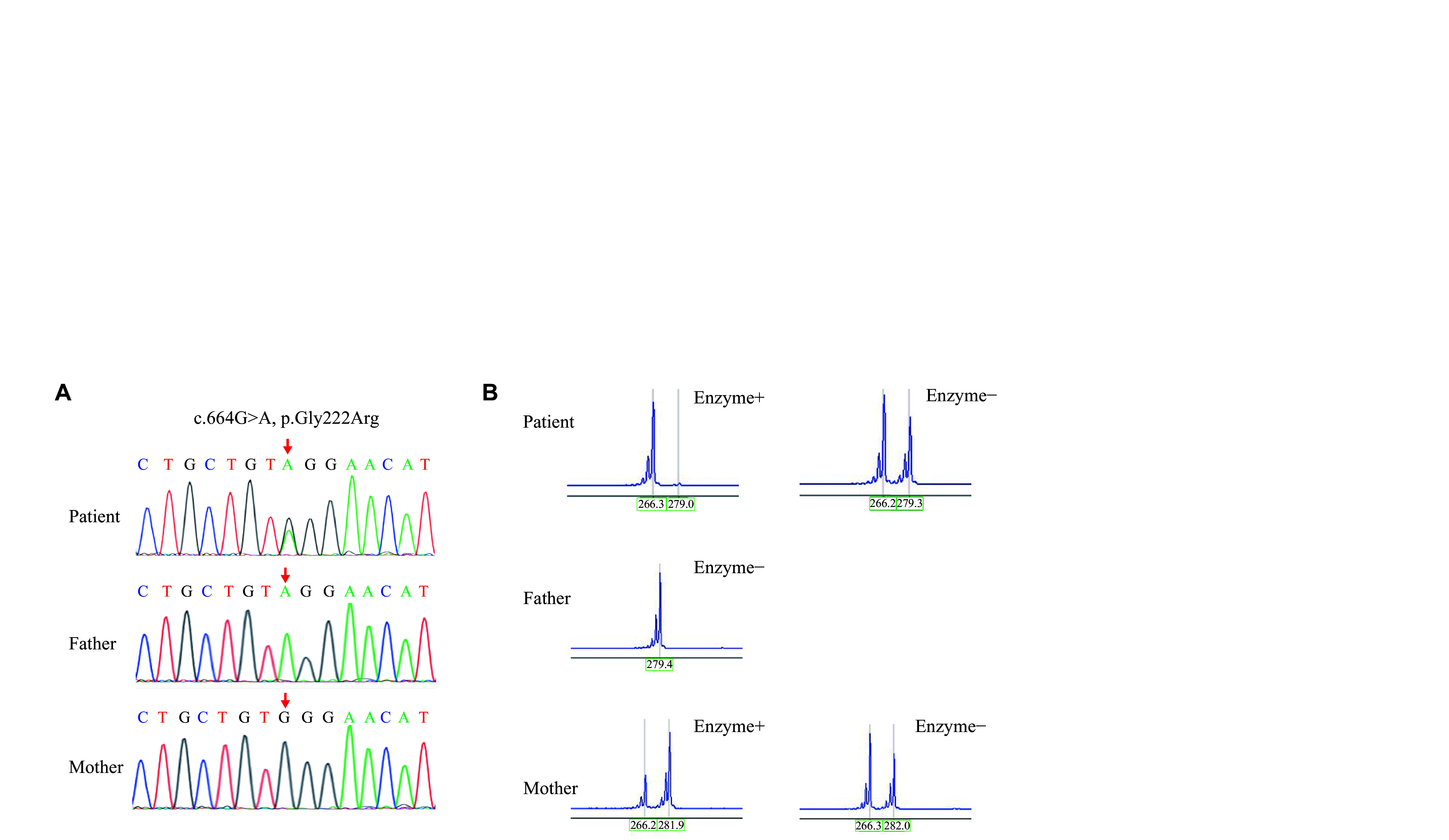
Clinical features, genetic mutations, and capillary electrophoresis results in female pediatric patients. A: The Sanger sequencing of the *CLCN5* gene of patient 4 and her parents showed that the patient had a novel *CLCN5* heterozygous variation in exon 6 (NM_000084.4: c.664G>A), while her father was hemizygous for the same mutation at the same site, and the mother was wild-type. B: The X-chromosome inactivation (XCI) analysis by the human androgen receptor gene (HUMARA) assay.

In addition, P4, the only female patient in the current study, piqued our interest. She inherited one normal allele from her mother and one variant allele from her father. As we know, the *CLCN5* gene is located on the X chromosome. Therefore, female heterozygous carriers are usually asymptomatic in theory, but this female patient developed LMWP. We conducted a further XCI test to verify if the allele from her healthy mother was inactivated. Indeed, we found that the patient had an extremely skewed pattern of XCI, with 97% of her maternal allele being inactivated in mature blood cells (***Fig. 2***).

### Correlation between mutation type and the clinical phenotype of NP

The current study not only included 21 children from our center but also summarized a total of 88 children with Dent disease who had NP, all of whom were male. All mutations were summarized as either truncating mutations (nonsense, splicing defects, and incomplete insertion or deletion resulting in a frameshift and truncated protein) or non-truncating mutations (missense mutation, small in-frame insertion or deletion). The correlation between mutation type and the clinical phenotype of NP was not statistically significant (***Table 3***). Therefore, the severity of the NP phenotype in children with Dent disease may not be determined based on the type of mutation.

**Table 3:** Correlation between genotype and phenotype in 88 children with Dent disease

Characteristics	NTMs (*n*=40)	TMs (*n*=48)	*P*
NP	35	46	0.2378
Non-NP	5	2	
Data were analyzed by Chi-square test. Abbreviations: NTMs, non-truncating mutations; TMs, truncating mutations; NP, nephrotic-range proteinuria.			

### Effect of novel variant on cellular expression

**Figure 3:**
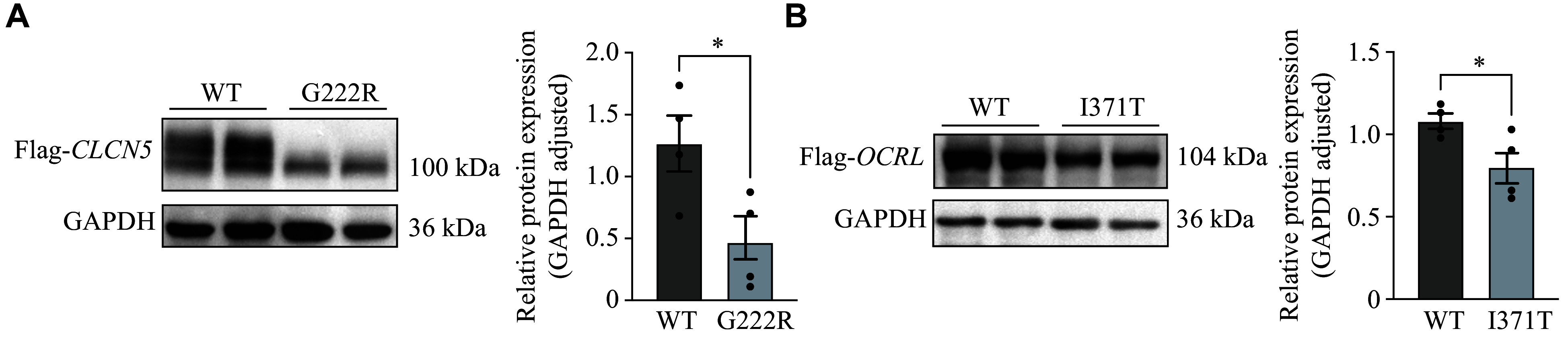
Functional characterization of novel gene variants in HEK293 cells. A: HEK293 cells were transfected with *CLCN5*-WT or the mutant (G222R) plasmid (2 μg) for 4 h and harvested after a 24-h culture for Western blotting. The CLC-5 protein levels in HEK293 cells transiently expressing the *CLCN5*-WT or mutant (G222R) were immunoblotted with the anti-FLAG antibody and quantified. B: HEK293 cells were transfected with the *OCRL*-WT or mutant (I371T) plasmid (2 μg) for 4 h and harvested after a 24-h culture for Western blotting. The OCRL1 protein levels in HEK293 cells transiently expressing *OCRL*-WT or the mutant (I371T) were immunoblotted with the anti-FLAG antibody and quantified. The samples were derived from the same experiment, and the gels/blots were processed in parallel. Data are presented as mean ± standard error of the mean (*n* = 4). ^*^P < 0.05 by unpaired Student's *t*-test. Full-length blots/gels images are presented in ***Supplementary Fig. 2*** (available online). Abbreviations: WT, wild-type; CLC-5, chloride voltage-gated channel 5; OCRL1, OCRL inositol polyphosphate-5-phosphatase.

In the current study, we identified two novel missense mutations: one in the *CLCN5* gene (G222R) and the other in the *OCRL* gene (I371T). Western blotting analyses showed that in the HEK293 cells, the *CLCN5*-WT protein was expressed as a double band with an apparent molecular weight distributed around 100 kDa. The variant protein (G222R) appeared as a lower protein band, and its expression levels were significantly decreased (***Fig. 3A***). The *OCRL*-WT protein was expressed as a single band with an apparent molecular weight of 104 kDa. The variant protein (I371T) also appeared with significantly reduced expression levels compared to wild-type OCRL1, while it exhibited a similar molecular weight (***Fig. 3B***). Therefore, the missense variants G222R and I371T showed significant inconsistencies in protein expression, compared with the WT protein expression in HEK 293 cells.

**Figure 4:**
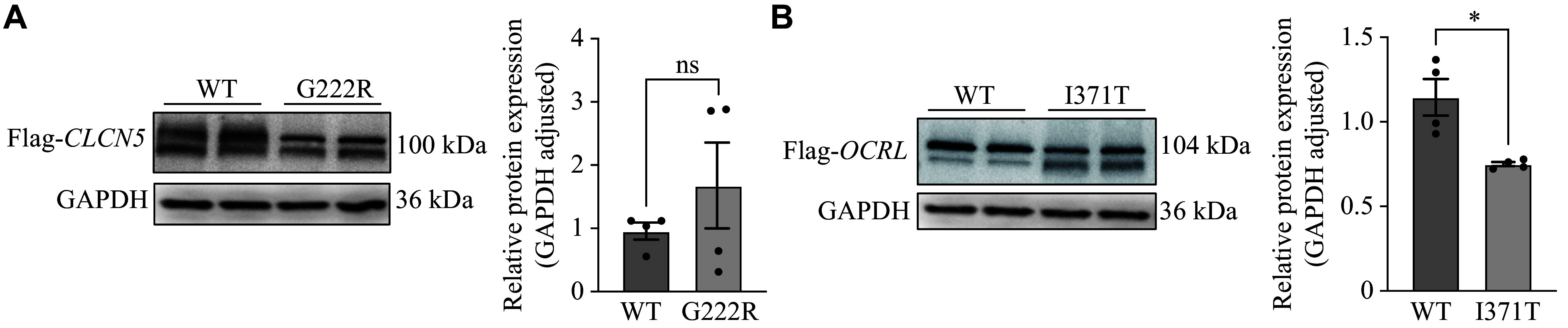
Functional characterization of novel gene variants in HK-2 cells. A: HK-2 cells were transfected with *CLCN5*-WT or the mutant (G222R) plasmid (2 μg) for 4 h and harvested after 24-h of culture for Western blotting. The CLC-5 protein levels in HK-2 cells transiently expressing the *CLCN5*-WT or mutant (G222R) were immunoblotted with the anti-FLAG antibody and quantified. B: HK-2 cells were transfected with the *OCRL*-WT or mutant (I371T) plasmid (2 μg) for 4 h and harvested after 24-h of culture for Western blotting. The OCRL1 protein levels in HK-2 cells transiently expressing *OCRL*-WT or the mutant (I371T) were immunoblotted with the anti-FLAG antibody and quantified. The samples were derived from the same experiment, and the gels/blots were processed in parallel. Data are presented as mean ± standard error of the mean (*n* = 4). ^*^P < 0.05 by unpaired Student's *t*-test. Abbreviations: WT, wild-type; CLC-5, chloride voltage-gated channel 5; OCRL1, OCRL inositol polyphosphate-5-phosphatase; ns, not significant.

We also investigated the protein expression profiles of CLC-5 and OCRL1 in HK-2 cells transfected with either WT or mutant constructs: the G222R missense variant and the I371T missense variant. Western blotting analysis demonstrated a significantly reduced expression of the I371T mutant protein (104 kDa) compared with its wild-type counterpart. In contrast, the G222R mutant (100 kDa) exhibited comparable expression levels to those of WT CLC-5 across all transfected cell groups (***Fig. 4***).

## Discussion

All 21 patients in the current study exhibited varying degrees of proteinuria, of whom 14 developed NP without hypoalbuminemia and edema, which could initially be interpreted as glomerular proteinuria. However, further urine protein testing revealed predominantly LMWP, suggesting a tubular disease. Eleven of these 14 patients had variants in the *CLCN5* gene, while three had variants in the *OCRL* gene. Patient 20 (P20) was initially misdiagnosed with nephrotic syndrome and then was treated with several immunosuppressive agents, including steroids and cyclophosphamide, without any effect. This not only imposed a heavy economic burden on families but, more importantly, might have led to serious adverse effects such as infections and impaired growth and development. NP in Dent disease cases abroad is not uncommon^[11–12]^. In the current study, we observed 14 children with NP and conducted a literature search on variant sites in these children. We found that 10 variants were previously reported, of which the most frequently reported missense variant, S244L in *CLCN5*, was investigated by Grand *et al*^[13]^ who found it to significantly reduce the ionic current. However, it did not affect its cellular location, and the mechanisms by which this variant causes NP were not investigated. The remaining previously reported variant sites also did not show an association between NP and the variant site. Because functional data are lacking for most of the variants, variants that lead to messenger RNA decay or premature termination (such as nonsense, frameshift, or gross deletion) of the resultant OCRL1 or CLC-5 proteins were classified as severe, while missense variants were classified as moderate^[14]^. Furthermore, van Berkel *et al*^[12]^ found that the prevalence of NP was comparable to that of patients with severe variants. However, we did not find any correlation between NP and variant severity in the current study.

Among the clinical signs of Dent disease, glomerular disease is often underestimated, and some cases are mistaken for being the result of tubular injury. The primary renal pathological manifestations of Dent disease include FSGS, FGGS, mesangial proliferative glomerulonephritis, and MCD. Renal tubular damage is predominantly characterized by tubular atrophy and interstitial fibrosis. Segmental fusion of podocytes is observed in the majority of Dent disease patients. In 2016, Wang *et al*^[11]^ proposed that podocyte damage was correlated with renal function, suggesting that it was an early indicator of renal impairment in Dent disease. Ceol *et al*^[15]^ first confirmed the expression of CLC-5 in human podocytes in 2012, indicating a potential link between podocyte damage and CLC-5 dysfunction. In 2020, Preston *et al*^[16]^ demonstrated that OCRL1 was expressed in adult kidney tissues and the glomeruli of zebrafish larvae; in cultured podocytes, the expression levels of OCRL1 were found to be correlated with those of the adapter proteins IPIP27A and CD2AP, which were crucial for maintaining the podocyte slit diaphragm. In 2023, Priante *et al*^[17]^ reported that abnormal CLC-5 function altered podocyte function directly by disrupting the actin cytoskeleton or indirectly by impairing the human nephrin cycle. Additionally, Dent disease may present with variations in basement membrane thickness, with thickening being the most prevalent finding. More recent reports^[18–19]^ indicated that patients with Dent disease might exhibit diffuse basement membrane thinning, which was a rare manifestation correlated with thin basement membrane nephropathy or Dent disease subtype. However, none of these have been confirmed to date.

The mechanisms underlying glomerulosclerosis in patients with Dent disease remain unclear, and no standardized biomarker has been identified for its diagnosis. Further investigation into the glomerular podocytes and the basement membrane is warranted. While glomerulosclerosis is found in almost two-thirds of the biopsies of Dent disease, it also suggests that damage to the glomeruli may be responsible for the appearance of NP in Dent disease^[20–21]^. Moreover, Edvardsson *et al*^[7]^ recommended a diagnostic flowchart for Dent disease in 2013, in which an elevated LMWP was a key clue to the diagnosis of Dent disease. Therefore, it is recommended to perform an accurate urine protein electrophoresis in children with newly diagnosed proteinuria, especially those without a significant decline in plasma albumin. This will help clarify the urinary protein composition and nature, and, if necessary, assist in further genetic testing for proper diagnosis.

To date, Dent disease has been reported to be caused by more than 350 *CLCN5* variants and 370 *OCRL* variants. The majority of *CLCN5* variants are missense and frameshift (35% and 31%, respectively), followed by nonsense variants (16%), splicing variants (10%), and large deletions (4%). Moreover, different disease-causing variants have been described in *OCRL*, such as insertions, deletions, splicing, and missense variants^[22]^. In the current study, we identified four novel variants, including one missense variant, the *CLCN5* missense variant (G222R). The missense variant was predicted to have deleterious consequences based on pathogenicity and conservation analyses. According to the ACMG guidelines, deletions of exons 2–4 are pathogenic, whereas deletions of exon 5 are likely pathogenic.

The G222R variant is located around the F helix, which is an important component of the chloride (Cl^−^) selectivity filter composed of the D, F, N, and R helices^[23–25]^. Therefore, the variant site may alter the corresponding ionic current. Pusch *et al*^[26]^ reported five missense variants in the F helix that did not affect cell surface expression but shifted the protein's subcellular localization from the Golgi to the endoplasmic reticulum. In the current study, we found that all variant sites in the *OCRL* gene were located within the 5-phosphatase catalytic domain coding region. This 5-phosphatase domain is the catalytic region and is involved in the phosphorylation of phosphoinositide^[27]^. To better understand the molecular mechanisms of the novel variants in our patient cohort, we evaluated the effect of the missense variant on CLC-5 and OCRL1 expression. The novel mutant (p.G222R in *CLCN5*) did not affect the CLC-5 protein levels in HK-2 cells but significantly reduced them in HEK293 cells. Conversely, the novel mutant (p.I371T in *OCRL*) affected protein expression in both HEK293 and HK-2 cells. Multiple studies have demonstrated that mutants of *CLCN5* and *OCRL* influence protein expression and impact ionic currents. Therefore, it is essential to perform more complex experiments, such as animal models and current assays, to elucidate the pathogenetic mechanisms involved^[1,3,25,28]^. Despite certain limitations, our findings regarding the multiple changes in CLC-mediated Cl^−^/H^+^ shifts caused by disease-related variants underscore the need for future studies to further enhance our understanding of the molecular genetic basis of Dent disease.

Dent disease mainly affects males below the age of 10 and is extremely rare in females. In females, the disease typically presents with normal or mild LMWP symptoms^[23]^. One study^[29]^ reported that the HUMARA assay revealed extreme XCI skewing, which may affect disease onset or severity in female carriers of X-linked disorders. However, no study has yet established a correlation between the XCI pattern and the onset of Dent disease in female carriers. Female mammals may compensate for the X-linked gene product by inactivating one of the two X chromosomes during embryonic development^[30]^. The copy of the X chromosome that undergoes inactivation is stochastically determined; thus, heterozygous female patients with X-linked recessive disorders usually present with mild or no clinical phenotype, compared with male patients. Moreover, studies of female patients with Dent disease have shown that most female patients do not have typical symptoms except for LMWP^[31–32]^. This suggests that the prevalence of Dent disease in this population may be largely underestimated because of mild symptoms in females. This should not be overlooked clinically in a female child with isolated LMWP.

In conclusion, the current study suggests that LMWP may be the primary clinical manifestation of some children with Dent disease. Dent disease is rare in female children, and the skewed XCI pattern may contribute to the phenotypic diversity. Because of the low incidence of Dent disease, it may be easily misdiagnosed clinically. When a diagnosis of FGGS or FSGS is suggested by NP or renal biopsy, it is important to analyze the nature of the proteinuria to avoid unnecessary steroid and immunosuppressant therapy. Routine urine tests for children aged three years in Japan have significantly reduced the age of disease onset; hence, more aggressive clinical screening and genetic testing strategies should be adopted.

## SUPPLEMENTARY DATA

Supplementary data to this article can be found online.
