# Supplementary material for: Phenotype and genotype analyses of 21 Chinese patients with Dent disease
Source: J Biomed Res. 2025 May 27;39(4):356–66. doi: 10.7555/JBR.38.20240183 (PMC12336408; doi:10.7555/JBR.38.20240183)
Supplement: Supplementary file 1 — Supplementary data to this article can be found online. [file jbr-39-4-356-Supplementary.pdf]

Available online at www.jbr-pub.org.cn

Open Access at PubMed Central

Journal of Biomedical Research, 2025 39(4): 1–4

JBR

Original Article

Phenotype and genotype analyses of 21 Chinese patients with Dent disease

Ruochen Che[1,△], Yuwen Cai[1,△], Wei Zhou[2], Sanlong Zhao[1,✉], Songming Huang[1,3,✉]

[1]*Department of Nephrology, Children's Hospital of Nanjing Medical University, Nanjing, Jiangsu 210029, China;*
[2]*Nanjing Key Laboratory of Pediatrics, Children's Hospital of Nanjing Medical University, Nanjing, Jiangsu 210029, China;*
[3]*Jiangsu Key Laboratory of Pediatrics, Nanjing Medical University, Jiangsu 211166, Nanjing, China.*

Supplementary Table 1 **Sequences of primers used for amplification and sequencing of the coding regions of the *CLCN5* and *OCRL* genes**

Genes	Forward (5′-3′)	Reverse (5′-3′)
CLCN5		
Exon 2	TCATCTGATAGTTTAAGGGCCCG	ATTTCCTAACACTTACCCATGTGC
Exon 3	GGTCTCTATTCTCCAGTGATTTG	ACTTACGGGAGAGACATACCAC
Exon 4	CCGAGATTCAGTTTAACTTTGGC	TCCGCAATTGCTGTGAGCACAG
Exon 5	TGTCTCACTGAAGCTGTGTAGGC	CTTAGATGGCCTCAGGATCTG
Exon 6	TAGCCTTGTTGACTTCCTTAGTC	CAAAAAGAGACTATTAAGGCCATTC
Exon 7	GTTCAGTAACTACTAACATTTCTTC	CACAAAGCATGCACATGCTGTAC
Exon 8-1	GACTGAGTTTGCTTTCTCACCTTC	CTTGGAGGAGTCCAGAAGGCC
Exon 8-2	CAGCCATCACTGCCATCCTG	ATACCTGGGCTCTGCCCTCC
Exon 9	TCACTAACCATCTATTGGTTTCTC	TCTTCTGTTGTATGTCACCTGGG
Exon 10-1	GAGAACTCTGAATCGTCTCCATTG	ACGGATGTGGGCATCATAGATG
Exon 10-2	CATGACAAGCAAGTGGGTGGC	GTTCCACAGTGGATTCCACTATC
Exon 11	TCCATCTTCAATTTGTTTTTTCCTTC	GCATCCTCTTTCTCCCACAATTC
Exon 12	TGAAAAGGACTGAGGAGGACAAG	GGTACCAGTTAATACAACATATCC
OCRL		
Exon 1	GGAGCTGTTCCTCAAACGA	CTCTCTGCTCGGCCTCTG
Exon 2	GGGTGGAAGACCCCCTTC	ACCTGGACCTGAACCTGTTG
Exon 3	CAGTGGCTGTTCTTTGATGC	TTCGATATAACCCTTCAGCATTC
Exon 4	AGGAGTAGTGACTGAGAGAAGCAG	CATGTTTTTGGGAAACAGTGC
Exon 5	TTGTGATCTGGACCTTCTTCTG	TCAGAATTCCACTCCCGTTG
Exon 6	GCCCCTGCATATAAGGAATG	CATCACCATATTTGGCCTGAC
Exon 7	TTTTTCCCCGTTTGACTTTG	TTTTCACGTGGAAGCCTAGC
Exon 8	CATATTTGCCTTGTAGGAGAAT	CAACAGGCCACTGTCTGTT
Exon 9	CCTTTGTATGGAAGCGAAAAG	CACAAATTTGACCCGCAAG
Exon 10	GACAGGAGGTAGCCAGAGAT	TGGAATAACTCCCGGTGAG
Exon 11	CGTGGGACATTAGAAATGTGG	GCTACCCCACCTTTGTTTCC

△These authors contributed equally to this work.

✉Corresponding authors: Sanlong Zhao and Songming Huang, Department of Nephrology, Children's Hospital of Nanjing Medical University, 72 Guangzhou Road, Nanjing, Jiangsu 210029, China. E-mails: slzhao1981@163.com (Zhao) and smhuang@njmu.edu.cn (Huang).

Received: 25 June 2024; Revised: 26 February 2025; Accepted: 06 March 2025; Published online: 27 May 2025

CLC number: R692.6, Document code: A

The authors reported no conflict of interests.

This is an open access article under the Creative Commons Attribution (CC BY 4.0) license, which permits others to distribute, remix, adapt and build upon this work, for commercial use, provided the original work is properly cited.

© 2025 by Journal of Biomedical Research.

https://doi.org/10.7555/JBR.38.20240183

Supplementary Table 1 **Sequences of primers used for amplification and sequencing of the coding regions of the *CLCN5* and *OCRL1* genes** (continued)

Genes	Forward (5′-3′)	Reverse (5′-3′)
Exon 12	GGTGAGTTACTTTGGAAATGAGC	GCCACTACTCAAAGGCTACAGG
Exon 13	AGTGGTGAGTGAGCCCTTATC	CAGTAAGACGTTTCCATCACTCC
Exon 14	TGGCTTATCAACCTGATTATCTC	AGCCCCTTACCAATATGGAG
Exon 15	CCCTGATCTAAACCAGTGTGG	GGAACATTTATGGCTTGCTG
Exon 16	TCCAAGGGAGATGAGCTAGAAG	CAACTGGAATGGAGGCAGTC
Exon 17	CTATGGCATTTGCACACCTG	ATCACCAGCAGAGACAATGG
Exon 18	TTCCCACTGGAGGTTTTCC	TGAAAATTAAATGTAAAAGAGGTT
Exon 19	GCATGACCAGAATTTGAAGG	CGTGAGGTGTTGTGATTTCC
Exon 20	GCTGCTCTTCTGATCCTTGG	GAGAAACAAGTGGATAGTCAGTGG
Exon 21	TCTGTGTGGCCTTTCTCCTG	ATGCAGACCCATCCTACCAG
Exon 22	TCCACCTGTTTTTCTCACTGC	CCTGGGGACAAGGACTATTG
Exon 23	GCCCTGAGGTTTTGCTTAGG	GCAATAGACCCTTCCTGTGG

Supplementary Table 2 **Clinical and genetic analysis of 21 Dent disease patients**

Patient	Sex	Age of onset	Microscopic hematuria	Hypercalciuria	NP	LMWP	Nephrocalcinosis	Gene	Nucleotide change	Type of mutation	Amino acid change
P1	Male	1 Y 2 M	−	+	+	+	+	*CLCN5*	c.731C>T	Missense	p.S244L
P2	Male	5 Y 5 M	+	−	+	+	+	*CLCN5*	Exon5 del	Deletion	Exon deletion
P3	Male	1 Y 1 M	−	+	NA	+	+	*CLCN5*	c.731C>T	Missense	p.S244L
P4	Female	5 Y 2 M	−	−	−	+	−	*CLCN5*	c.664G>A	Missense	p.G222R
P5	Male	3 Y 6 M	−	−	−	+	+	*CLCN5*	c.1942C>T	Nonsense	p.R648X,99
P6	Male	1 Y	−	−	+	+	+	*CLCN5*	c.1909C>T	Nonsense	p.R637X,110
P7	Male	1 Y 9 M	+	−	+	+	−	*CLCN5*	c.429C>A	Nonsense	p.Y143X,604
P8	Male	2 Y 8 M	−	−	+	+	+	*CLCN5*	Exon6-10del	Deletion	Exon deletion
P9	Male	12 Y	−	+	+	+	+	*CLCN5*	c.1619C>A	Missense	p.A540D
P10	Male	8 M	−	−	+	+	−	*CLCN5*	c.569_c.590delCTCTGGTTA TCAAAACCATCAC	Frameshift	p.T190Tfs*10
P11	Male	4 Y 2 M	−	−	−	+	−	*CLCN5*	c.194G>A	Missense	p.G65E
P12	Male	6 Y	−	−	+	+	−	*CLCN5*	exon2-4del	Deletion	Exon deletion
P13	Male	10 M	−	−	+	+	−	*CLCN5*	c.1546C>T	Missense	p.R516W
P14	Male	1 Y 2 M	−	+	+	+	+	*CLCN5*	c.1898G>T	Missense	p.G633V
P15	Male	13 Y11 M	+	−	−	+	+	*CLCN5*	Exon5-11del	Deletion	Exon deletion
P16	Male	5 Y 10 M	+	+	+	+	+	*CLCN5*	c.1432_c.1433insT	Frameshift	p.Y478fs*11
P17	Male	9 Y 3 M	+	+	+	+	−	*OCRL1*	c.821T>C	Missense	p.I274T
P18	Male	11 Y 8 M	−	−	−	+	−	*OCRL1*	c.1112T>C	Missense	p.I371T
P19	Male	7 Y 10 M	−	+	−	+	−	*OCRL1*	c.1196 T>C	Missense	p.F399S
P20	Male	1 M	−	−	+	+	−	*OCRL1*	c.723_c.724insT	Frameshift	p.F242Ffs*15
P21	Male	13 Y 8 M	−	+	+	+	−	*OCRL1*	c.1436 A>G	Missense	p.Y479C

Abbreviation: P: patient; NA, not applicable; Y, year; M, month; +, positive; −, negative.

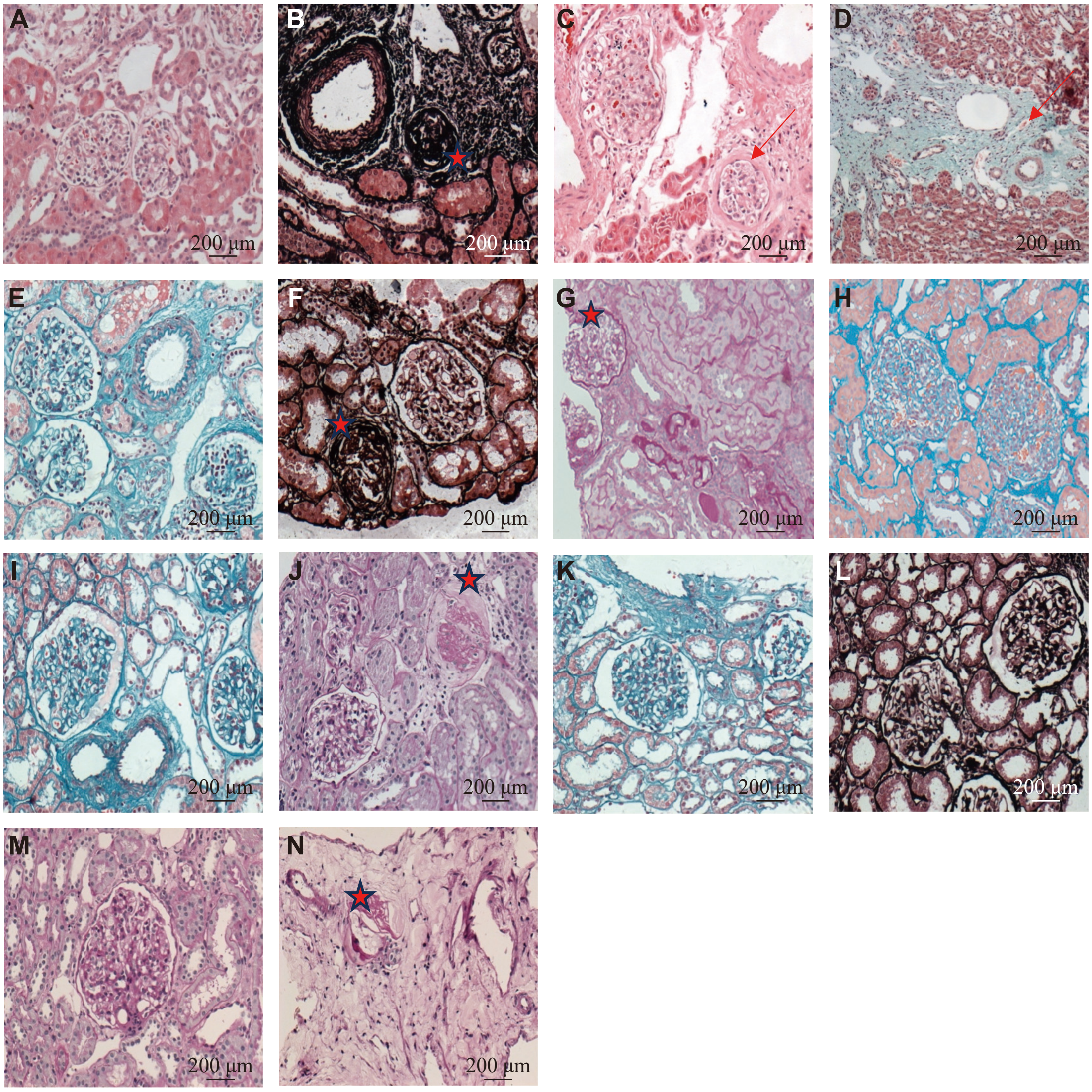

Supplementary Fig. 1 **Renal histopathologic features in Dent disease.** A and B: The renal histopathologic features of P6. Among 53 glomeruli, two glomeruli exhibit global sclerosis (star), while the remaining glomeruli show mild renal lesions with mild focal tubular atrophy and interstitial fibrosis. HE staining, ×200 (A) and PASM staining, ×200 (B). C and D: The renal histopathologic features of P9. Six glomeruli present with mild hyperplasia of mesangial matrix, fibrosis of Bowman's capsule wall in one glomerulus (arrow), and focal tubular atrophy accompanied by focal interstitial fibrosis (arrow). HE staining, ×200 (C) and Masson staining, ×100 (D). E and F: The renal histopathological features observed in P14. Among 22 glomeruli, one glomerulus exhibits global sclerosis (star), while the remaining glomeruli show mild lesions, with mild focal tubular atrophy accompanied by focal interstitial fibrosis. Masson staining, ×200 (E) and PASM staining, ×200 (F). G and H: The renal histopathological features observed in P15. 42 glomeruli present, with four glomeruli showing global sclerosis (star), while the remaining glomeruli exhibit mild lesions. PAS staining, ×200 (G) and Masson staining, ×200 (H). I and J: The renal histopathological features observed in P18. 34 glomeruli present, with one glomerulus showing global sclerosis (star), while the remaining glomeruli exhibit mild lesions with no significant tubulointerstitial lesions. Masson staining, ×200 (I) and PAS staining, ×200 (J). K and L: The renal histopathological features exhibited by patient P19. 16 glomeruli present, with mild glomerular lesions and no significant lesions in renal tubules and interstitium. Masson staining, ×200 (K) and PASM staining, ×200 (L). M and N: The renal histopathological features exhibited by patient P21. 10 glomeruli present, with one glomerulus exhibiting global sclerosis (star), while the remaining glomeruli show mild lesions with no significant tubulointerstitial lesions. PAS staining, ×200 (M and N). Abbreviations: P, patient; HE staining, hematoxylin-eosin staining; PASM, periodic acid silver methenamine; PAS, periodic acid-Schiff.

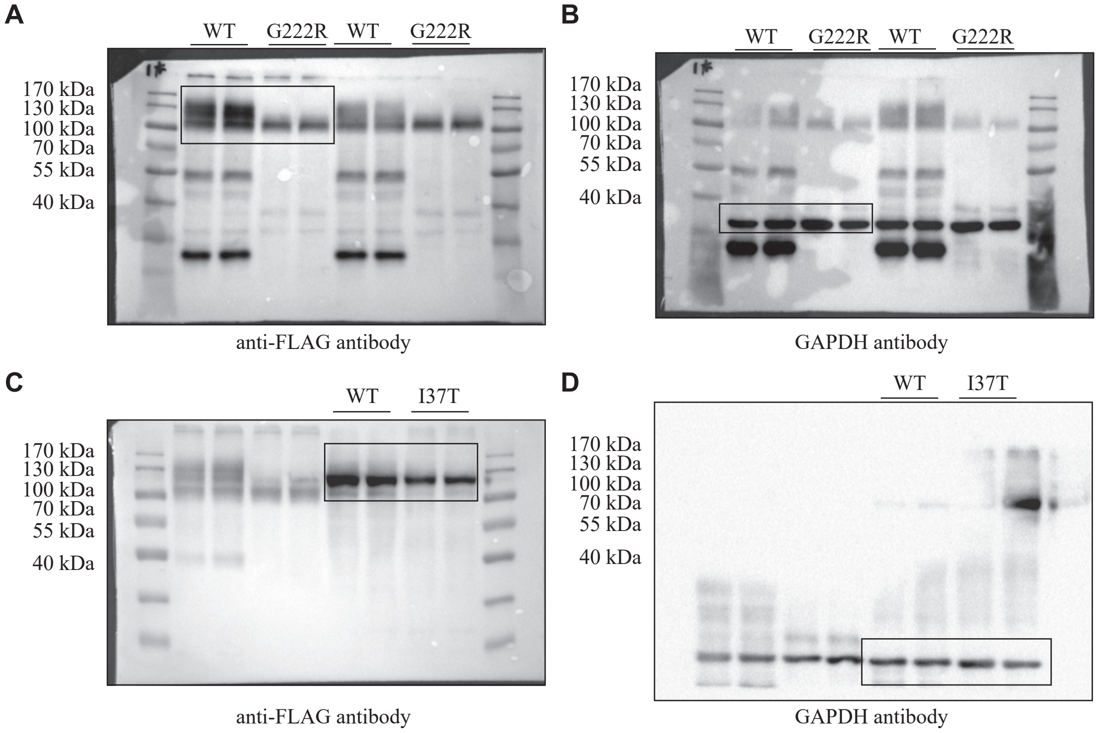

Supplementary Fig. 2 **Full-length blots/gels of *Fig. 3*.**
